# Sleep Duration and Cancer in the NIH-AARP Diet and Health Study Cohort

**DOI:** 10.1371/journal.pone.0161561

**Published:** 2016-09-09

**Authors:** Fangyi Gu, Qian Xiao, Lisa W. Chu, Kai Yu, Charles E. Matthews, Ann W. Hsing, Neil E. Caporaso

**Affiliations:** 1 Division of Cancer Epidemiology and Genetics, National Cancer Institute, Bethesda, MD, United States of America; 2 Cancer Prevention Institute of California, Fremont, CA, United States of America; 3 Stanford Cancer Institute, Palo Alto, CA, United States of America; 4 Department of Health Research and Policy, School of Medicine, Stanford University, Stanford, CA, United States of America; National Health Research Institutes, TAIWAN

## Abstract

**Background:**

Very few studies have examined sleep duration in relation to cancer incidence with the exception of breast cancer.

**Methods:**

We assessed the associations between sleep duration and incidences of total and 18 site-specific cancers in the NIH-AARP Health and Diet Study cohort, with 173,327 men and 123,858 women aged 51–72 years at baseline. Self-reported sleep duration categories were assessed via questionnaire. We used multivariable Cox proportional hazards regression to estimate hazard ratios (HR) and 95% confidence intervals (CI), using 7–8 hours/night as the reference.

**Results:**

We observed a significantly increased risk of stomach cancer among male short sleepers (multivariable HR_5-6 vs. 7–8 hours_ = 1.29; 95%CI: 1.05, 1.59; *P*_trend_ = 0.03). We also observed suggestive associations in either short or long sleepers, which did not reach overall significance (*P*_trend_ >0.05), including increased risks in male short sleepers for cancers of head and neck (HR_<5vs.7-8 hours_ = 1.39; 95%CI:1.00–1.95), bladder (HR_5-6vs.7-8 hours_ = 1.10; 95%CI:1.00–1.20), thyroid (HR_<5 vs. 7–8 hours_ = 2.30; 95%CI:1.06, 5.02), Non-Hodgkin Lymphoma (NHL) (HR_5-6vs.7-8 hours_ = 1.17; 95%CI:1.02–1.33), and myeloma (HR_<5vs.7-8 hours_ = 2.06; 95%CI:1.20–3.51). In women, the suggestive associations include a decreased total cancer risk (HR_<5vs.7-8 hours_ = 0.9; 95%CI:0.83–0.99) and breast cancer risk (HR_<5vs.7-8 hours_ = 0.84; 95%CI:0.71–0.98) among short sleepers. A decreased ovarian cancer risk (HR_≥ 9 vs. 7–8 hours_ = 0.50; 95%CI:0.26–0.97) and an increased NHL risk (HR_≥ 9 vs. 7–8 hours_ = 1.45; 95%CI:1.00–2.11) were observed among long sleepers.

**Conclusion:**

In an older population, we observed an increased stomach cancer risk in male short sleepers and suggestive associations with short or long sleep duration for many cancer risks in both genders.

## Introduction

On average, adults sleep 7–8 hours per day [1]. Both shorter and longer sleep duration, compared to 7 or 8 hours per night, have been associated with increases in obesity [2–4], diabetes [5,6], and all-cause mortality. Biologically, sleep disruption might also link with cancer through its impact on the neuroendocrine-immune system complex that regulates cell proliferation, immune defense, energy metabolism, and adaption to everyday stresses [7]. Night shift work has been associated with elevated risks of multiple cancers and was categorized as a potential carcinogen [8]. However, with the exception of breast cancer, where inconsistent results were reported [9–17], relatively few population studies have examined sleep duration and diverse other cancer sites. These studies reported an increased risk of colorectal cancer among longer sleepers [18,19], a decreased risk of prostate cancer among longer sleepers [20], and non-significant decreased risk of thyroid cancer among short sleepers [21] and non-significant decreased risk of endometrial cancer among longer sleepers [22].

We examined night-time sleep duration and the incidence of total and 18 site-specific cancers in the NIH-AARP Health and Diet Study cohort among 173,327 men and 123,858 women.

## Materials and Methods

### Study population

The NIH-AARP Diet and Health Study, described in detail previously [23], was established in 1995–1996 to evaluate association of diet and health. A total of 567,169 of 3.5 million members of AARP (formerly known as the American Association of Retired Persons), aged 50–71, completed baseline questionnaires, who resided in one of six states (California, Florida, Pennsylvania, New Jersey, North Carolina, and Louisiana) or in two metropolitan areas (Atlanta, Georgia and Detroit, Michigan). A risk factor questionnaire (RFQ), which assessed sleep duration and medical histories, was sent to the baseline cohort one year after enrollment, and was completed satisfactorily by 337,076 respondents. After the exclusion of those died or moved out of the study area before RFQ scan, 334,905 subjects remained in the RFQ cohort. We additionally excluded 1,532 subjects due to missing sleep information, 23,925 with prevalent cancers, 4,029 without diagnosis date, and 9,332 who completed questionnaire by proxy. A total of 297,185 (173,327 men and 123,858 women) remained in the analytical cohort. The study was approved by the National Cancer Institute Special Studies Institutional Review Board. Return of the questionnaire was considered to be informed consent.

### Cohort follow-up

Participants were followed annually for change of address by linkage to the National Change of Address maintained by the U.S. Postal Service, through receipt of U.S. Postal Service processing of undeliverable mail, from other address change update services, and directly from participants who report address changes in response to study mailings. Vital status was ascertained by annual linkage of the cohort to the Social Security Administration Death Master File (SSA DMF) on deaths in the U.S., follow-up searches of the National Death Index for subjects that match to the SSA DMF, cancer registry linkage, questionnaire responses, and responses to other mailings. There was no loss of follow up for mortality.

### Exposure and covariate assessment

Demographic, diet and life style, sleep, and disease history information was collected in the baseline and risk factors questionnaires (distributed one year after baseline). Information of sleep duration and napping were collected through the risk factor questionnaire by asking “during a typical 24-hour period over the past 12 months, how many hours did you spend sleeping at night” and “napping during the day”? The sleep duration question had four predetermined categorical responses (<5, 5–6, 7–8, and ≥9 hours) The risk factor questionnaire also asked information on detailed medical history including medication use and prostate cancer screening using a prostate specific antigen (PSA) test. In the baseline questionnaire, we asked about demographic characteristics, current body weight and height, medical history, family history of cancer, and lifestyle factors including frequency of vigorous physical activity that lasted at least 20 minutes, smoking status, time since quitting smoking, and smoking dose. Dietary intake was assessed with a self-administered 124 item food frequency questionnaire (FFQ) [24].

### Identification of cancer cases

During follow-up from 1995 to 2006, incident cases of cancer were identified by probabilistic linkage with a cancer registry databases from the original 8 states and 3 additional states (Arizona, Nevada and Texas) where the AARP participants moved to. The cancer registries for this cohort are estimated to be 95% complete within two years of cancer incidents and are certified by the North American Association of Central Cancer Registries (NAACCR) for meeting the highest standard of data quality. Our cancer ascertainment methods has been validated by linking a subset of our cohort (n = 12,000) to all eight cancer registries and comparing the data to self-reports and subsequent medical record confirmation of incident cancer, which demonstrated that about 90% of all cancer cases were validly identified using cancer registries [25].

Incident cancer cases were invasive and consisted only of the first malignant neoplasm diagnosed during the follow-up period if multiple cancers had been diagnosed in the same participant. We defined cancers using the Surveillance Epidemiology and End Results (SEER) incidence site recode and the International Classification of Diseases for Oncology code (3rd ed.): oral, head and neck (C000-C009, C019-C119, C129-C140, C142-C148, C300-C301, C310-C329, C339-C349, C381-C384, C388, C390, C398, and C399), esophagus (C150-C159), stomach (C160-C169), colorectal (C180-C189, C199, C209, and C260), liver (C220 and C221), pancreas (C250-C259), lung (C340-C349), bladder (C670-C679), kidney (C649 and C659), thyroid (C739), brain (C710-C719), breast (C500-C509), ovarian (C569), endometrial (C540-C549, and C559), and prostate (C619). Non-Hodgkin lymphoma (NHL), leukemia, and myeloma were also defined by SEER definition, with the Mid-level SEER cancer site group 35, 37–39 and 36 respectively. Total cancer included those cancers listed above, skin cancer excluding basal/squamous, other miscellaneous cancers, and unspecified cancers.

### Statistical analyses

We used Cox proportional hazards regression, to obtain hazard ratios (HR) and 95% confidence intervals (CI) of total and site-specific cancers (as well as cancers grouped by anatomical system) for each sleep duration category at night (7–8 hour as reference). Person years of follow-up was calculated from the date of risk questionnaire completion until the date of cancer diagnosis, death, move out of the registry areas, or end of follow-up (Dec. 2006), whichever occurred first. We examined the linear trend of sleep duration on cancer incidence by modeling a numeric value for each sleep category (1 for <5 hours, 2 for 5–6 hours, 3 for 7–8 hours and 4 for ≥9 hours). We comprehensively adjusted for all cancer risk/protective factors available in our dataset that could serve as confounders: age, gender, napping, race, education, marital status, self-reported health, family history of cancer, smoking (former/current/never, as well as dose and years after quitting), physical activity, sitting time, diabetes, hypertension, body mass index (BMI), Nonsteroidal anti-inflammatory drug (NSAID) use, alcohol drinking, intakes of fruits and vegetables, wholegrain, total fat, red meat and total calories. For female reproductive cancer (breast, endometrial and ovary), we additionally adjusted for post-menopausal hormone use, menopausal status, number of live child birth, oral contraception use, hysterectomy and oophorectomy. For prostate cancer, we additionally adjusted for Prostate-Specific Antigen (PSA) screening. We also performed analyses in men and women separately. We evaluated and confirmed the proportional hazards assumption for the main exposures by including interaction terms with time and using the Wald χ^2^ procedure to test if coefficients equaled zero.

To assess reverse causation, we did sensitivity analyses by excluding cancer diagnosis within 2 years. For cancers with large enough samples (lung, breast, prostate, and colon), we also did sensitivity analyses by excluding diabetes, and participants with poor health condition from our data, and conducted stratified analyses by BMI (<25, ≥25 kg/m^2^), physical activity (<3, ≥3 hours/week) and napping (yes, no) for these cancer sites. Since diabetes and BMI could be the results of sleep deprivation [4,5,26], we also did sensitivity analyses to check if these factors mediated the association between sleep duration and cancer outcomes, by removing these two factors from the multivariable model. To avoid potential over adjustments of non-confounders and/or intermediates, we additionally removed physical activity, sedentary behavior, hypertension, or any dietary variables. For breast cancer, we did further sensitivity analyses by restricting to women who never used postmenopausal hormone.

We used SAS software version 9.3 to conduct the analyses. All P values are two sided with 0.05 as the significance level.

## Results

During a total of 11 years of follow up, we observed 38,879 total incident cancers, and the numbers of site specific cancer cases ranged from 339 (liver cancer) to 14,044 (prostate cancer); with over 4,000 cases of colorectal, breast and lung cancer. At baseline, we identified 8,743 (2.9%), 94,122 (31.7%), 184,176 (62.0%) and 10,144 (3.4%) participants who reported <5 hours, 5–6 hours, 7–8 hours and ≥9 hours of sleep per night, respectively (Table 1). Baseline characteristics of our study population for each gender were showed in Table 1.

**Table 1:** Characteristics of study population at baseline by sleep duration in men and women.

	Sleep duration in men				Sleep duration in women			
	<5 hr	5–6 hr	7–8 hr	≥9 hr	<5 hr	5–6 hr	7–8 hr	≥9 hr
No. of subjects (%)	4302 (2.5)	53099 (30.6)	109976 (63.5)	5950 (3.4)	4441 (3.6)	41023 (33.1)	74200 (59.9)	4194 (3.4)
Age, yr, mean (SD)	62.4 (5.4)	62.4 (5.4)	63.2 (5.2)	64.3 (4.9)	62.7 (5.4)	62.5 (5.3)	62.6 (5.3)	62.9 (5.3)
White, non-Hispanic, %	87.6	92.1	96.0	96.6	84.2	89.0	94.4	94.6
College and postcollege, %	31.1	43.6	50.1	46.8	19.7	28.0	34.8	34.9
Married, %	76.1	83.6	87.0	83.2	64.2	59.2	51.6	50.8
Self-reported health, excellent, %	11.1	16.3	19.8	16.1	9.7	15.6	19.8	16.6
Family history of cancer, %	29.2	28.5	29.4	30.5	34.8	31.9	31.8	31.2
Current smoker, %	14.7	10.7	8.5	9.3	16.1	14.4	12.4	11.5
Former smoker, %	54.1	56.9	57.7	59.0	35.8	38.0	40.0	41.9
Physical activity > = 5 times/wk, %	20.5	22.5	24.7	22.9	24.3	23.8	23.4	20.9
Sitting < 3 hr/d, %	24.3	19.9	17.9	17.1	27.4	23.4	21.2	19.9
No napping, %	32.0	40.8	48.8	44.4	50.5	45.9	39.9	43.7
Body mass index, kg/m2, mean (SD)	28.7 (5.9)	27.5 (4.4)	27.2 (4.5)	27.2 (4.5)	28.8 (7.3)	27.1 (6.2)	26.3 (5.6)	26.9 (6.0)
Personal history of diabetes, %	15.7	10.7	8.7	12.8	13.6	7.7	5.7	8.1
Personal history of hypertension, %	47.5	41.5	39.4	46.5	47.7	39.2	35.1	40.5
NSAID use, %	58.1	72.5	73.2	70.6	63.2	67.9	70.0	67.7
Alcohol, g/d, mean (SD)	19.2 (61.3)	16.1 (42.7)	18.1 (42.0)	31.1 (65.3)	4.6 (18.2)	5.2 (17.6)	6.5 (17.5)	10.0 (28.7)
Fruits and vegetables, serving/kcal, mean (SD)	3.6 (1.9)	3.7 (1.7)	3.6 (1.6)	3.3 (1.6)	4.5 (2.3)	4.5 (2.0)	4.4 (1.9)	4.2 (1.9)
Whole grains, serving/kcal, mean (SD)	0.6 (0.5)	0.7 (0.5)	0.7 (0.5)	0.6 (0.5)	0.6 (0.5)	0.7 (0.5)	0.7 (0.4)	0.7 (0.5)
Total fat, g/kcal, mean (SD)	0.3 (0.1)	0.3 (0.1)	0.3 (0.1)	0.3 (0.1)	0.3 (0.1)	0.3 (0.1)	0.3 (0.1)	0.3 (0.1)
Red meat, g/kcal, mean (SD)	39.1 (24.4)	37.5 (22.5)	37.2 (21.7)	38.0 (22.2)	29.7 (20.6)	29.1 (19.4)	28.9 (18.8)	29.7 (20.3)
Total energy, kcal/d, mean (SD)	2277.8 (1380.6)	2040 (1012)	2014 (913)	2199 (1092)	1785 (1195)	1600 (790)	1560 (688)	1681 (1020)
PSA test in past 3 years, %	63.4	69.9	72.8	70.2				
Menopausal hormone therapy use, ever, %					42.6	51.1	56.5	58.0
Post-menopausal, %					94.5	93.7	93.4	92.3
Nulliparous, %					14.5	14.7	15.7	17.0

Hr: hour, SD:standard deviation, kg/m2:kilogram per meter square, g: gram, kcal: kilocalorie

We observed a significantly increased risk of stomach cancer among men of short sleep duration (HR_5-6 vs. 7–8 hours_ = 1.29; 95%CI: 1.05, 1.59; P-trend = 0.03), based on the fully adjusted model (multivariable model 2). We did not find other associations reached overall statistical significance level, but suggestive associations were observed for many cancers with either short or long sleepers in both genders. In men, these suggestive associations include the increased risks among short sleepers for head and neck cancer (fully-adjusted HR_<5 vs. 7–8 hours_ = 1.39; 95%CI: 1.00, 1.95), bladder cancer (fully-adjusted HR_5-6 vs. 7–8 hours_ = 1.10; 95%CI:1.00, 1.20), Non-Hodgkin Lymphoma (NHL) (fully-adjusted HR_5-6 vs. 7–8 hours_ = 1.17; 95%CI:1.02,1.33), and myeloma (fully-adjusted HR_<5 vs. 7–8 hours_ = 2.06; 95%CI:1.20, 3.51) (Table 2). Removing BMI and diabetes from the full adjustment strengthened the association for thyroid cancer (HR_<5 vs. 7–8 hours_ = 2.30; 95%CI:1.06, 5.02 in the Multivariable model 1), but did not influence the associations for other cancer sites.

**Table 2:** Association between night time sleep duration and cancer incidence in man.

	Hazard Ratio (95% Confidence Interval)				
	Sleep at night <5 hr	5–6 hr	7–8 hr	≥9 hr	*P*_trend_
All cancers (N = 32778)	799	9791	20963	1225	
Age adjusted	1.07 (1.00, 1.15)	1.01 (0.99, 1.04)	ref	1.06 (1.00, 1.12)	*0*.*48*
Multivariable 1	1.02 (0.95, 1.09)	1.00 (0.98, 1.03)	ref	1.03 (0.97, 1.09)	*0*.*99*
Multivariable 2 (Fully adjusted)	1.02 (0.95, 1.10)	1.00 (0.98, 1.03)	ref	1.02 (0.96, 1.08)	*0*.*91*
Head and Neck cancer (N = 1044)	38	337	627	42	
Age adjusted	1.68 (1.21, 2.33)	1.15 (1.01, 1.32)	ref	1.23 (0.90, 1.68)	*0*.*01*
Multivariable 1	1.38 (0.99, 1.93)	1.11 (0.97, 1.27)	ref	1.08 (0.79, 1.48)	*0*.*06*
Multivariable 2	1.39 (1.00, 1.95)	1.12 (0.98, 1.28)	ref	1.05 (0.77, 1.44)	*0*.*05*
Esophageal Cancer (N = 417)	11	136	252	18	
Age adjusted	1.23 (0.67, 2.24)	1.17 (0.95, 1.44)	ref	1.30 (0.80, 2.09)	*0*.*38*
Multivariable 1	0.97 (0.53, 1.78)	1.11 (0.90, 1.37)	ref	1.12 (0.69, 1.81)	*0*.*62*
Multivariable 2	0.97 (0.53, 1.80)	1.11 (0.90, 1.37)	ref	1.10 (0.68, 1.79)	*0*.*58*
Stomach Cancer (N = 409)	11	153	233	12	
Age adjusted	1.33 (0.73, 2.44)	1.43 (1.17, 1.76)	ref	0.92 (0.52, 1.65)	*0*.*001*
Multivariable 1	1.06 (0.58, 1.96)	1.33 (1.08, 1.63)	ref	0.87 (0.49, 1.56)	*0*.*02*
Multivariable 2	0.98 (0.53, 1.81)	1.29 (1.05, 1.59)	ref	0.84 (0.47, 1.50)	*0*.*03*
Colorectal Cancer (N = 2895)	75	880	1816	124	
Age adjusted	1.16 (0.92, 1.46)	1.05 (0.97, 1.14)	ref	1.23 (1.03, 1.48)	*0*.*3*
Multivariable 1	1.09 (0.86, 1.37)	1.04 (0.96, 1.13)	ref	1.16 (0.97, 1.40)	*0*.*49*
Multivariable 2	1.03 (0.82, 1.3)	1.03 (0.94, 1.11)	ref	1.12 (0.93, 1.35)	*0*.*88*
Liver Cancer (N = 256)	10	80	153	13	
Age adjusted	1.85 (0.98, 3.51)	1.14 (0.87, 1.49)	ref	1.53 (0.87, 2.70)	*0*.*35*
Multivariable 1	1.27 (0.66, 2.42)	1.01 (0.77, 1.33)	ref	1.32 (0.75, 2.33)	*0*.*98*
Multivariable 2	1.11 (0.58, 2.14)	0.97 (0.74, 1.27)	ref	1.25 (0.70, 2.21)	*0*.*82*
Pancreatic Cancer (N = 657)	24	193	417	23	
Age adjusted	1.64 (1.09, 2.47)	1.01 (0.85, 1.20)	ref	0.99 (0.65, 1.51)	*0*.*23*
Multivariable 1	1.52 (1.01, 2.31)	1.00 (0.84, 1.18)	ref	0.95 (0.62, 1.45)	*0*.*32*
Multivariable 2	1.50 (0.99, 2.28)	0.98 (0.83, 1.17)	ref	0.95 (0.62, 1.45)	*0*.*39*
Lung Cancer (N = 3454)	99	1065	2148	142	
Age adjusted	1.31 (1.07, 1.61)	1.09 (1.01, 1.17)	ref	1.18 (1.00, 1.4)	*0*.*03*
Multivariable 1	0.91 (0.74, 1.12)	0.99 (0.92, 1.07)	ref	0.99 (0.83, 1.17)	*0*.*6*
Multivariable 2	0.92 (0.75, 1.13)	1.00 (0.93, 1.08)	ref	0.96 (0.81, 1.14)	*0*.*65*
Prostate Cancer (N = 14044)	299	4076	9199	470	
Age adjusted	0.91 (0.81, 1.02)	0.96 (0.92, 0.99)	ref	0.93 (0.85, 1.02)	*0*.*04*
Multivariable 1	0.95 (0.84, 1.06)	0.96 (0.93, 1.00)	ref	0.95 (0.87, 1.04)	*0*.*14*
Multivariable 2	0.96 (0.86, 1.08)	0.97 (0.94, 1.01)	ref	0.96 (0.87, 1.05)	*0*.*27*
Bladder Cancer (N = 2167)	49	688	1355	75	
Age adjusted	1.04 (0.78, 1.38)	1.12 (1.02, 1.23)	ref	0.98 (0.78, 1.24)	*0*.*03*
Multivariable 1	0.96 (0.72, 1.28)	1.11 (1.01, 1.21)	ref	0.92 (0.73, 1.16)	*0*.*06*
Multivariable 2	0.95 (0.72, 1.27)	1.10 (1.00, 1.20)	ref	0.92 (0.73, 1.16)	*0*.*07*
Kidney Cancer (N = 927)	29	292	564	42	
Age adjusted	1.44 (0.99, 2.08)	1.11 (0.97, 1.28)	ref	1.37 (1.00, 1.88)	*0*.*25*
Multivariable 1	1.25 (0.86, 1.83)	1.06 (0.92, 1.22)	ref	1.35 (0.98, 1.84)	*0*.*72*
Multivariable 2	1.15 (0.79, 1.68)	1.03 (0.89, 1.19)	ref	1.29 (0.94, 1.77)	*0*.*92*
Thyroid Cancer (N = 154)	7	51	90	6	
Age adjusted	2.15 (1.00, 4.64)	1.21 (0.86, 1.70)	ref	1.24 (0.54, 2.84)	*0*.*13*
Multivariable 1	2.30 (1.06, 5.02)	1.21 (0.86, 1.71)	ref	1.36 (0.59, 3.11)	*0*.*14*
Multivariable 2	2.09 (0.95, 4.60)	1.16 (0.82, 1.64)	ref	1.31 (0.57, 3.00)	*0*.*19*
Brain Cancer (N = 322)	6	87	220	9	
Age adjusted	0.75 (0.33, 1.68)	0.84 (0.66, 1.08)	ref	0.76 (0.39, 1.48)	*0*.*29*
Multivariable 1	0.76 (0.33, 1.71)	0.85 (0.66, 1.09)	ref	0.77 (0.40, 1.51)	*0*.*31*
Multivariable 2	0.78 (0.34, 1.76)	0.85 (0.66, 1.09)	ref	0.78 (0.40, 1.53)	*0*.*32*
Non-Hodgkin Lymphoma (n = 1084)	24	358	664	38	
Age adjusted	1.01 (0.67, 1.52)	1.16 (1.02, 1.32)	ref	1.04 (0.75, 1.45)	*0*.*09*
Multivariable 1	1.02 (0.68, 1.54)	1.18 (1.03, 1.34)	ref	1.04 (0.75, 1.44)	*0*.*06*
Multivariable 2	1.00 (0.66, 1.51)	1.17 (1.02, 1.33)	ref	1.05 (0.75, 1.45)	*0*.*07*
Leukemia (N = 708)	15	223	439	31	
Age adjusted	0.97 (0.58, 1.63)	1.11 (0.95, 1.31)	ref	1.26 (0.88, 1.81)	*0*.*66*
Multivariable 1	0.95 (0.57, 1.60)	1.11 (0.94, 1.30)	ref	1.26 (0.87, 1.82)	*0*.*74*
Multivariable 2	0.93 (0.55, 1.56)	1.10 (0.94, 1.30)	ref	1.23 (0.85, 1.78)	*0*.*73*
Myeloma (N = 349)	15	108	213	13	
Age adjusted	1.99 (1.18, 3.36)	1.10 (0.87, 1.39)	ref	1.11 (0.63, 1.94)	*0*.*09*
Multivariable 1	2.03 (1.19, 3.45)	1.11 (0.88, 1.40)	ref	1.11 (0.63, 1.95)	*0*.*1*
Multivariable 2	2.06 (1.20, 3.51)	1.13 (0.89, 1.43)	ref	1.10 (0.63, 1.93)	*0*.*06*

Cox proportional hazard model was used to calculate hazard ratios.

Multivariable 1 removed body mass index and diabetes from covariance adjustment of Multivariable 2

Multivariable 2 adjusted for age, napping, race, education, marital status, self-reported health, family history of cancer, smoking (former/current/never, as well as dose and years after quitting), physical activity, sitting time, diabetes, hypertension, body mass index, NSAID use, alcohol drinking, intakes of fruits and vegetables, wholegrain, total fat, red meat and total calories.

In women (Table 3), the suggestive associations based on the fully adjusted model include a decreased risks of total cancer (HR_<5 vs. 7–8 hours_ = 0.9; 95%CI: 0.83, 0.99) and breast cancer (HR_<5 vs. 7–8 hours_ = 0.84; 95%CI: 0.71, 0.98) among short sleepers, as well as a decreased ovarian cancer risk (HR_≥ 9 vs. 7–8 hours_ = 0.50; 95%CI: 0.26, 0.97), and an increased NHL risk (HR_≥ 9 vs. 7–8 hours_ = 1.45; 95%CI: 1.00, 2.11) among long sleepers.

**Table 3:** Association between night time sleep duration and cancer incidence in women.

	Hazard Ratio (95% Confidence Interval)				
	Sleep at night<5 hr	5–6 hr	7–8 hr	≥9 hr	*P*_trend_
All cancers (N = 16101)	526	5287	9735	553	
Age adjusted	0.92 (0.84, 1.01)	0.98 (0.95, 1.02)	ref	1.01 (0.93, 1.10)	*0*.*07*
Multivariable 1	0.91 (0.83, 0.99)	0.99 (0.96, 1.02)	ref	0.98 (0.90, 1.06)	*0*.*19*
Multivariable 2 (Fully adjusted)	0.9 (0.83, 0.99)	0.99 (0.95, 1.02)	ref	0.96 (0.89, 1.05)	*0*.*17*
Head and Neck cancer (N = 292)	13	91	174	14	
Age adjusted	1.27 (0.72, 2.24)	0.95 (0.73, 1.22)	ref	1.43 (0.83, 2.46)	*0*.*67*
Multivariable 1	1.12 (0.63, 1.98)	0.9 (0.7, 1.16)	ref	1.34 (0.77, 2.31)	*0*.*39*
Multivariable 2	1.16 (0.65, 2.07)	0.92 (0.71, 1.19)	ref	1.28 (0.74, 2.21)	*0*.*51*
Esophageal Cancer (N = 66)	2	25	37	2	
Age adjusted	0.92 (0.22, 3.82)	1.22 (0.74, 2.03)	ref	0.95 (0.23, 3.94)	*0*.*58*
Multivariable 1	0.73 (0.17, 3.12)	1.18 (0.71, 1.98)	ref	0.77 (0.19, 3.22)	*0*.*69*
Multivariable 2	0.78 (0.18, 3.35)	1.22 (0.72, 2.04)	ref	0.78 (0.19, 3.24)	*0*.*62*
Stomach Cancer (N = 113)	3	41	67	2	
Age adjusted	0.76 (0.24, 2.41)	1.11 (0.75, 1.64)	ref	0.52 (0.13, 2.13)	*0*.*59*
Multivariable 1	0.62 (0.19, 1.98)	1.03 (0.7, 1.53)	ref	0.5 (0.12, 2.05)	*0*.*93*
Multivariable 2	0.58 (0.18, 1.88)	1.05 (0.7, 1.55)	ref	0.49 (0.12, 2.00)	*0*.*95*
Colorectal Cancer (N = 1507)	50	504	893	60	
Age adjusted	0.95 (0.72, 1.26)	1.02 (0.92, 1.14)	ref	1.18 (0.91, 1.53)	*0*.*68*
Multivariable 1	0.88 (0.66, 1.18)	0.99 (0.89, 1.11)	ref	1.16 (0.89, 1.51)	*0*.*31*
Multivariable 2	0.86 (0.64, 1.15)	0.99 (0.89, 1.11)	ref	1.13 (0.87, 1.47)	*0*.*29*
Liver Cancer (N = 83)	4	36	41	2	
Age adjusted	1.66 (0.59, 4.63)	1.59 (1.02, 2.49)	ref	0.86 (0.21, 3.54)	*0*.*04*
Multivariable 1	1.31 (0.46, 3.71)	1.46 (0.93, 2.3)	ref	0.79 (0.19, 3.26)	*0*.*12*
Multivariable 2	1.22 (0.43, 3.49)	1.41 (0.89, 2.22)	ref	0.79 (0.19, 3.29)	*0*.*17*
Pancreatic Cancer (N = 408)	16	135	243	14	
Age adjusted	1.12 (0.68, 1.86)	1.01 (0.82, 1.24)	ref	1.01 (0.59, 1.74)	*0*.*8*
Multivariable 1	0.96 (0.57, 1.60)	0.96 (0.78, 1.19)	ref	0.96 (0.56, 1.65)	*0*.*79*
Multivariable 2	0.97 (0.58, 1.63)	0.97 (0.78, 1.2)	ref	0.97 (0.57, 1.67)	*0*.*82*
Lung Cancer (N = 3454)	84	765	1235	70	
Age adjusted	1.16 (0.93, 1.45)	1.12 (1.03, 1.23)	ref	1 (0.79, 1.27)	*0*.*01*
Multivariable 1	0.90 (0.72, 1.13)	1.05 (0.95, 1.15)	ref	0.92 (0.72, 1.17)	*0*.*63*
Multivariable 2	0.91 (0.73, 1.15)	1.05 (0.96, 1.15)	ref	0.91 (0.71, 1.16)	*0*.*59*
Breast Cancer (N = 5919)	162	1920	3648	189	
Age adjusted	0.76 (0.65, 0.89)	0.95 (0.9, 1.01)	ref	0.93 (0.80, 1.07)	*0*.*007*
Multivariable 1	0.84 (0.72, 0.99)	1.00 (0.95, 1.06)	ref	0.90 (0.78, 1.04)	*0*.*67*
Multivariable 2	0.84 (0.71, 0.98)	1.00 (0.94, 1.05)	ref	0.89 (0.77, 1.03)	*0*.*69*
Ovarian Cancer (N = 515)	13	175	318	9	
Age adjusted	0.70 (0.40, 1.21)	1.00 (0.83, 1.2)	ref	0.50 (0.26, 0.98)	*0*.*86*
Multivariable 1	0.77 (0.44, 1.36)	1.05 (0.87, 1.26)	ref	0.50 (0.26, 0.97)	*0*.*69*
Multivariable 2	0.78 (0.45, 1.37)	1.05 (0.87, 1.27)	ref	0.50 (0.26, 0.97)	*0*.*37*
Endometrial Cancer (N = 1030)	47	304	635	44	
Age adjusted	1.26 (0.94, 1.70)	0.87 (0.76, 0.99)	ref	1.24 (0.91, 1.68)	*0*.*19*
Multivariable 1	1.30 (0.96, 1.76)	0.90 (0.78, 1.03)	ref	1.17 (0.86, 1.59)	*0*.*48*
Multivariable 2	1.20 (0.88, 1.62)	0.88 (0.76, 1.01)	ref	1.11 (0.82, 1.52)	*0*.*52*
Bladder Cancer (N = 382)	15	117	235	15	
Age adjusted	1.09 (0.64, 1.83)	0.90 (0.72, 1.13)	ref	1.12 (0.67, 1.89)	*0*.*48*
Multivariable 1	0.98 (0.58, 1.67)	0.88 (0.70, 1.10)	ref	1.07 (0.64, 1.81)	*0*.*32*
Multivariable 2	1.03 (0.60, 1.75)	0.88 (0.70, 1.10)	ref	1.09 (0.64, 1.84)	*0*.*39*
Kidney Cancer (N = 337)	11	124	192	10	
Age adjusted	0.98 (0.53, 1.80)	1.17 (0.93, 1.47)	ref	0.92 (0.49, 1.75)	*0*.*28*
Multivariable 1	0.74 (0.40, 1.38)	1.07 (0.85, 1.34)	ref	0.88 (0.47, 1.67)	*0*.*96*
Multivariable 2	0.73 (0.39, 1.35)	1.06 (0.84, 1.33)	ref	0.85 (0.45, 1.62)	*0*.*92*
Thyroid Cancer (N = 192)	9	59	117	7	
Age adjusted	1.32 (0.67, 2.6)	0.91 (0.67, 1.24)	ref	1.08 (0.51, 2.32)	*0*.*92*
Multivariable 1	1.15 (0.58, 2.3)	0.87 (0.64, 1.2)	ref	1.06 (0.49, 2.28)	*0*.*65*
Multivariable 2	1.11 (0.55, 2.22)	0.87 (0.63, 1.19)	ref	1.05 (0.49, 2.25)	*0*.*6*
Brain Cancer (N = 158)	4	61	87	6	
Age adjusted	0.79 (0.29, 2.14)	1.27 (0.91, 1.76)	ref	1.23 (0.54, 2.81)	*0*.*53*
Multivariable 1	0.84 (0.31, 2.32)	1.31 (0.94, 1.83)	ref	1.23 (0.54, 2.81)	*0*.*39*
Multivariable 2	0.80 (0.29, 2.21)	1.29 (0.92, 1.79)	ref	1.24 (0.54, 2.86)	*0*.*47*
Non-Hodgkin Lymphoma (N = 628)	14	205	379	30	
Age adjusted	0.63 (0.37, 1.08)	0.98 (0.83, 1.16)	ref	1.41 (0.97, 2.04)	*0*.*07*
Multivariable 1	0.66 (0.38, 1.12)	0.99 (0.83, 1.17)	ref	1.46 (1.01, 2.12)	*0*.*08*
Multivariable 2	0.64 (0.37, 1.10)	0.98 (0.82, 1.16)	ref	1.45 (1.00, 2.11)	*0*.*07*
Leukemia (N = 256)	9	91	144	12	
Age adjusted	1.07 (0.54, 2.09)	1.14 (0.88, 1.49)	ref	1.48 (0.82, 2.67)	*0*.*08*
Multivariable 1	1.03 (0.52, 2.05)	1.15 (0.88, 1.50)	ref	1.45 (0.80, 2.61)	*0*.*77*
Multivariable 2	1.00 (0.50, 1.99)	1.14 (0.87, 1.49)	ref	1.42 (0.79, 2.57)	*0*.*79*
Myeloma (N = 170)	9	52	106	3	
Age adjusted	1.45 (0.73, 2.86)	0.89 (0.64, 1.24)	ref	0.50 (0.16, 1.57)	*0*.*57*
Multivariable 1	1.26 (0.63, 2.53)	0.85 (0.6, 1.19)	ref	0.49 (0.16, 1.54)	*0*.*84*
Multivariable 2	1.18 (0.58, 2.36)	0.83 (0.59, 1.16)	ref	0.45 (0.14, 1.43)	*0*.*91*

Cox proportional hazard model was used to calculate hazard ratios

Multivariable 1 removed body mass index and diabetes from covariance adjustment of Multivariable 2

Multivariable 2 adjusted for age, napping, race, education, marital status, self-reported health, family history of cancer, smoking (former/current/never, as well as dose and years after quitting), physical activity, sitting time, diabetes, hypertension, body mass index, NSAID use, alcohol drinking, intakes of fruits and vegetables, wholegrain, total fat, red meat and total calories. For breast, ovarian and endometrial cancers model additionally adjusted for postmenopausal hormonal use, menopausal status, number of live child birth, oral contraception use, hysterectomy and oophorectomy.

Sensitivity analyses by removing physical activity, sedentary behavior, BMI, diabetes, hypertension, or any dietary variables did not change our results and conclusion (S1 Table). Removing cancers diagnosed within the first two years of follow-up did not change our observation materially (S2 Table). For prostate, breast, colorectal and lung cancer sites, which had cancer cases greater than 4,000, we also performed sensitivity analyses by excluding participants with diabetes and poor health conditions, as well as stratifying by BMI, physical exercise and napping. These analyses did not change our results materially (S3 Table).

## Discussion

In a large prospective US cohort of older people, we observed significantly increased risk of stomach cancer among male short sleepers in the fully adjusted model. In addition, we observed suggestive associations with short or long sleepers for other cancers, without reaching overall statistical significance. In men, these include potential increased risks among short sleepers for head and neck cancer, bladder cancer, thyroid cancer, Non-Hodgkin Lymphoma (NHL), and myeloma. In women, these include a suggestive decreased risks of total and breast cancer among short sleepers; a suggestive decreased ovarian cancer risk and a suggestive increased NHL risk among long sleepers. Given that we examined 18 cancer sites at the same time, none of our findings survives multiple comparison adjustment. Therefore these observed associations warrant further replication.

To our knowledge, this is the first study reporting the association between sleep and stomach cancer incidence. We found an increase in the risk of stomach cancer among men who slept 5–6 hours per night (HR_5-6 vs. 7–8 hours_ = 1.29; 95%CI: 1.05, 1.59), but no association was observed for those who slept fewer than five hours per night, a category with only 11 cancer cases. This association is less likely explained by reverse causation given that the results were unchanged after excluding patients diagnosed within the first two years of follow-up (HR_5-6 vs. 7–8 hours_ = 1.27; 95%CI:1.03, 1.56). Interestingly, we did not find an association with esophagus cancer, a site that shares many lifestyle risk factors with stomach cancer (http://sylvester.org/cancer/stomach-and-esophageal/education/definition). Biologically, this could be due to the disrupted immune-inflammation balance among the extreme short sleepers [27], which facilitates H-Pylori related carcinogenesis [28,29]. In western countries, Helicobacter pylori (H-Pylori) is the strongest risk factor for stomach cancer [30,31], but is not associated with risk of esophageal cancer [32]. However, this observation could be the result of chance given that we did not find similar pattern among those who slept < 5 hours per night, although results of this category based on 11 cancer cases has limited power, therefore replication is necessary to confirm our finding.

For breast cancer, there have been eight previous publications [9–17]; the results are mixed but most reported null results [9,12,14,15,17], including a meta-analyses [14] and a large prospective cohort study in US [12]. We observed a lower risk among short sleepers (HR_<5 vs. 7–8 hours_ = 0.80; 95%CI: 0.68,0.93).In contrast to previous reports, this seems to contradict the original hypothesis that short sleepers may have increased breast cancer risk through either less melatonin production [33], or impaired immune function [27]. However, our result is consistent with two prospective cohort studies [13] [16], both included older US women (on average age 62–63), with an over 92% postmenopausal rate. Consistent with the previous report [13], our subgroup analyses showed the pattern of lower risk of breast cancer remained in ER positive (HR_<5 vs. 7–8 hours_ = 0.87, 95%CI = 0.69–1.09) and PR positive (HR_<5 vs. 7–8 hours_ = 0.77, 95%CI = 0.59–1.01) breast cancer, but not in PR negative breast cancer (HR_<5 vs. 7–8 hours_ = 0.99, 95%CI = 0.69–1.42). The exact mechanism behind these findings is unclear, and may be related to estrogen levels, a breast cancer risk factor. Extremely short sleep (<5 hours) could be a symptom of low estrogen levels for post-menopausal women.

Very few studies have examined sleep duration and cancers other than breast: three for colorectal, one of each for prostate, thyroid and endometrial. In two prospective cohort studies, both reported increased colorectal cancer incidence in longer duration sleepers (≥9 hours) [18,19], but one was mainly restricted to individuals who snored or were overweight [19], and the other was restricted to hormone replacement therapy (HRT) users [18]. However none of them adjusted for comorbidity, therefore long sleepers could be an indicator of poor health condition. Among these two studies, one reported increased CRC among short sleepers (≤5 hours), the other did not, but with relatively small samples in this group. Our study did not observe increased CRC risk for sleepers of either short duration (HR_≤5 vs. 7–8 hours_ = 0.96; 95%CI: 0.80, 1.15) or long duration (HR_≥9 vs. 7–8 hours_ = 1.12, 95%CI: 0.97, 1.30).

A previous report using large prospectively collected data of postmenopausal women found a significant increase in thyroid cancer incidence among women with higher insomnia scores, but no association was observed with sleep duration[21]. We also found no association with thyroid cancer for women. But we observed a non-significant increase in thyroid cancer risk among men with sleep duration less than 5 hours (multivariable HR_<5 vs. 7–8 hours_ = 2.09; 95%CI: 0.95, 4.60). When we remove BMI, diabetes and hypertension from the model, the association was stronger (HR_<5 vs. 7–8 hours_ = 2.30; 95%CI: 1.06, 5.02).

There are multiple strengths to our study. To our knowledge, this is the first study to comprehensively examine sleep duration in relation to all major cancer types. The large sample size permits sufficient power to assess associations with major specific cancer sites. Given that the sleep duration data information was prospectively collected, any reporting error is independent of cancer status, and therefore will less likely be an issue. In addition, the NIH-AARP cohort allowed us to control for most potential confounders, which is also prospectively collected data. Sensitivity analyses were performed by excluding cases within first 2 years of follow-up to exclude reverse causation.

As is a general issue in studies of sleep and health outcomes, our study is limited by using a one-time self-reported sleep duration questionnaire. Self-reported sleep duration tends to report, on average, an hour longer than the estimation by actigraphy [34]; this will influence sleep categorization but not the results trajectory. Sleep duration may also be vulnerable to report errors associated with various behavioral and mental health conditions [35]; Also the reproducibility of the one-time report of sleep duration using the AARP questionnaire has not been evaluated. However, any reporting error due to previously mentioned reasons will be independent of cancer status, therefore may bias our results toward the null. In addition, the biological meaning of altered sleep duration is complex. For example, short sleep duration could be associated with good sleep quality, or may result from disturbed sleep due to disease conditions such as chronic pain [36] and esophageal reflux disease [37]. Sleep duration of ≥9 hours could reflect poor quality [36], or maybe due to a sleep phase delay [38]. The mixed origins of the sleep duration tails might partially explain the inconsistent associations between short sleep duration and breast and colorectal cancer. Future studies should collect sleep measures in more detail (both duration and quality as well as sleep phase and different reason for sleep duration tails) at multiple different life periods to take these biologically important components into account in relation to cancer development. Better characterizing sleep including duration in sleep stages using new technologies (i.e. smart phone apps) may provide improved sleep assessment in future studies. Another issue is the high exclusion rates of our study population, due to the low or poor response rate of the risk questionnaire. Compared to the excluded population, our analytical cohort is more likely to be female, have higher education, healthier life style and therefore are likely to have better sleep quality. However, as a prospective cohort study, the non-response at baseline is less likely to be dependent on their future cancer diagnosis (other than through the less healthy life styles mentioned above), and therefore may not influence the association. We did not collect past shiftwork exposure, a probable cancer risk factor that may also influence subsequent sleep quality after retirement [39]. Confounding by past shiftwork exposure, where it occur, may bias the results away from the null. We did not collect information of stress levels, a factor that may affect sleep duration and quality. However the correlation of stress and cancer risk is unclear, therefore confounding by stress is less of a concern.

In conclusion, we observed potential increased risks of several cancer sites among men of short sleep duration, and changed risks of several cancer sites in women of both short and long sleep duration in older population. Only the association of stomach cancer achieved overall statistical significance and no association survives multiple comparison adjustment. Further studies are warranted to replicate these findings.

## Supporting Information

S1 Table Sensitivity analyses checking potential over adjustment of covariates. Cox proportional hazard model was used to calculate hazard ratios. Multivariable 1removed physical activity, sedentary behavior, BMI, diabetes, hypertension, or any dietary variables from the multivariable 2. Multivariable 2 adjusted for age, gender, napping, race, education, marital status, self-reported health, family history of cancer, smoking (former/current/never, as well as dose and years after quitting), physical activity, sitting time, diabetes, hypertension, body mass index, NSAID use, alcohol drinking, intakes of fruits and vegetables, wholegrain, total fat, red meat and total calories. * For female cancers the multivariable model additionally adjusted for postmenopausal hormonal use, menopausal status, number of live child birth, oral contraception use, hysterectomy and oophorectomy. **For prostate cancer, we additionally adjusted for PSA screening.
(DOC)

S2 Table Sensitivity analyses by excluding cancer cases diagnosed within 2 years of enrollment for selected sites. Cox proportional hazard model was used to calculate hazard ratios. Model adjusted for age, gender, napping, race, education, marital status, self-reported health, family history of cancer, smoking (former/current/never, as well as dose and years after quitting), physical activity, sitting time, diabetes, hypertension, body mass index, NSAID use, alcohol drinking, intakes of fruits and vegetables, wholegrain, total fat, red meat and total calories. * For female cancers model additionally adjusted for postmenopausal hormonal use, menopausal status, number of live child birth, oral contraception use, hysterectomy and oophorectomy.
(DOC)

S3 Table Sensitivity analyses for cancer sites with case number > 4000. Cox proportional hazard model was used to calculate hazard ratios. Model adjusted for age, gender, napping, race, education, marital status, self-reported health, family history of cancer, smoking (former/current/never, as well as dose and years after quitting), physical activity, sitting time, diabetes, hypertension, body mass index, NSAID use, alcohol drinking, intakes of fruits and vegetables, wholegrain, total fat, red meat and total calories. For breast cancer, the model additionally adjusted for postmenopausal hormonal use, menopausal status, number of live child birth, oral contraception use, hysterectomy and oophorectomy. For prostate cancer, we additionally adjusted for PSA screening.
(DOC)
