# Supplementary material for: Sleep Duration and Cancer in the NIH-AARP Diet and Health Study Cohort
Source: PLoS One. 2016 Sep 9;11(9):e0161561. doi: 10.1371/journal.pone.0161561 (PMC5017779; doi:10.1371/journal.pone.0161561)
Supplement: S1 Table — Cox proportional hazard model was used to calculate hazard ratios. Multivariable 1removed physical activity, sedentary behavior, BMI, diabetes, hypertension, or any dietary variables from the multivariable 2. Multivariable 2 adjusted for age, gender, napping, race, education, marital status, self-reported health, family history of cancer, smoking (former/current/never, as well as dose and years after quitting), physical activity, sitting time, diabetes, hypertension, body mass index, NSAID use, alcohol drinking, intakes of fruits and vegetables, wholegrain, total fat, red meat and total calories. * For female cancers the multivariable model additionally adjusted for postmenopausal hormonal use, menopausal status, number of live child birth, oral contraception use, hysterectomy and oophorectomy. **For prostate cancer, we additionally adjusted for PSA screening. (DOC) [file pone.0161561.s001.doc]

**S1 Table. Sensitivity analyses checking potential over adjustment of covariates**

|  | **Hazard Ratio (95% Confidence Interval)** | | | |  |
| --- | --- | --- | --- | --- | --- |
|  | **Sleep at night <5 hr** | **5-6 hr** | **7-8 hr** | **≥9 hr** | ***P* _trend_** |
| All cancers |  |  |  |  |  |
| No. of cases | 1325 | 15078 | 30698 | 1778 |  |
| Age adjusted | 0.96 (0.90, 1.01) | 0.98 (0.96, 1.00) | ref | 1.04 (0.99, 1.09) | 0.00 |
| Multivariable 1 | 0.96 (0.91, 1.02) | 0.99 (0.97, 1.01) | ref | 1.02 (0.97, 1.07) | 0.10 |
| Multivariable 2 | 0.96 (0.91, 1.02) | 0.99 (0.97, 1.01) | ref | 1.00 (0.96, 1.05) | 0.23 |
| Head and Neck cancer |  |  |  |  |  |
| No. of cases | 51 | 428 | 801 | 56 |  |
| Age adjusted | 1.41 (1.06, 1.87) | 1.07 (0.95, 1.20) | ref | 1.26 (0.96, 1.65) | 0.21 |
| Multivariable 1 | 1.32 (0.99, 1.76) | 1.05 (0.94, 1.19) | ref | 1.18 (0.90, 1.55) | 0.31 |
| Multivariable 2 | 1.33 (1.00, 1.78) | 1.08 (0.95, 1.21) | ref | 1.10 (0.84, 1.45) | 0.17 |
| Esophageal Cancer |  |  |  |  |  |
| No. of cases | 13 | 161 | 28 | 20 |  |
| Age adjusted | 1.00 (0.57, 1.74) | 1.12 (0.92, 1.36) | ref | 1.24 (0.79, 1.95) | 0.62 |
| Multivariable 1 | 0.94 (0.54, 1.65) | 1.12 (0.92, 1.37) | ref | 1.12 (0.71, 1.76) | 0.56 |
| Multivariable 2 | 0.93 (0.53, 1.63) | 1.12 (0.92, 1.37) | ref | 1.06 (0.67, 1.68) | 0.51 |
| Stomach Cancer |  |  |  |  |  |
| No. of cases | 14 | 194 | 300 | 14 |  |
| Age adjusted | 1.04 (0.61, 1.78) | 1.31 (1.09, 1.57) | ref | 0.83 (0.48, 1.41) | 0.01 |
| Multivariable 1 | 0.93 (0.54, 1.61) | 1.26 (1.05, 1.52) | ref | 0.77 (0.45, 1.32) | 0.03 |
| Multivariable 2 | 0.87 (0.51, 1.50) | 1.24 (1.03, 1.49) | ref | 0.75 (0.44, 1.29) | 0.04 |
| Colorectal Cancer |  |  |  |  |  |
| No. of cases | 125 | 1384 | 2709 | 184 |  |
| Age adjusted | 1.03 (0.86, 1.23) | 1.03 (0.96, 1.10) | ref | 1.21 (1.04, 1.40) | 0.76 |
| Multivariable 1 | 0.99 (0.82, 1.18) | 1.02 (0.95, 1.09) | ref | 1.17 (1.00, 1.35) | 0.58 |
| Multivariable 2 | 0.96 (0.80, 1.15) | 1.02 (0.95, 1.09) | ref | 1.12 (0.97, 1.30) | 0.69 |
| Liver Cancer |  |  |  |  |  |
| No. of cases | 14 | 116 | 194 | 15 |  |
| Age adjusted | 1.61 (0.94, 2.77) | 1.21 (0.96, 1.52) | ref | 1.38 (0.81, 2.33) | 0.13 |
| Multivariable 1 | 1.23 (0.71, 2.14) | 1.09 (0.86, 1.37) | ref | 1.24 (0.73, 2.11) | 0.61 |
| Multivariable 2 | 1.13 (0.65, 1.97) | 1.07 (0.85, 1.35) | ref | 1.18 (0.70, 2.01) | 0.65 |
| Pancreatic Cancer |  |  |  |  |  |
| No. of cases | 40 | 328 | 660 | 37 |  |
| Age adjusted | 1.36 (0.99, 1.87) | 1.00 (0.88, 1.15) | ref | 1.00 (0.72, 1.39) | 0.34 |
| Multivariable 1 | 1.24 (0.89, 1.71) | 0.98 (0.86, 1.12) | ref | 0.95 (0.68, 1.32) | 0.59 |
| Multivariable 2 | 1.24 (0.90, 1.72) | 0.98 (0.86, 1.12) | ref | 0.95 (0.68, 1.33) | 0.58 |
| Lung Cancer |  |  |  |  |  |
| No. of cases | 183 | 1830 | 3383 | 212 |  |
| Age adjusted | 1.21 (1.04, 1.40) | 1.09 (1.03, 1.15) | ref | 1.12 (0.97, 1.28) | 0.01 |
| Multivariable 1 | 0.90 (0.77, 1.04) | 1.01 (0.95, 1.07) | ref | 0.98 (0.85, 1.12) | 0.70 |
| Multivariable 2 | 0.92 (0.79, 1.07) | 1.02 (0.96, 1.08) | ref | 0.95 (0.83, 1.09) | 0.88 |
| Bladder Cancer |  |  |  |  |  |
| No. of cases | 64 | 805 | 1590 | 90 |  |
| Age adjusted | 0.90 (0.70, 1.16) | 1.03 (0.94, 1.12) | ref | 1.00 (0.81, 1.23) | 0.93 |
| Multivariable 1 | 0.98 (0.76, 1.26) | 1.07 (0.98, 1.16) | ref | 0.95 (0.76, 1.17) | 0.20 |
| Multivariable 2 | 0.97 (0.76, 1.25) | 1.06 (0.97, 1.16) | ref | 0.94 (0.76, 1.17) | 0.20 |
| Kidney Cancer |  |  |  |  |  |
| No. of cases | 40 | 416 | 756 | 52 |  |
| Age adjusted | 1.17 (0.85, 1.61) | 1.10 (0.97, 1.24) | ref | 1.25 (0.94, 1.65) | 0.32 |
| Multivariable 1 | 1.06 (0.76, 1.46) | 1.07 (0.94, 1.2) | ref | 1.17 (0.88, 1.55) | 0.65 |
| Multivariable 2 | 1.00 (0.73, 1.38) | 1.05 (0.93, 1.18) | ref | 1.17 (0.88, 1.55) | 0.78 |
| Thyroid Cancer |  |  |  |  |  |
| No. of cases | 16 | 110 | 207 | 13 |  |
| Age adjusted | 1.68 (1.01, 2.80) | 1.04 (0.83, 1.31) | ref | 1.18 (0.67, 2.06) | 0.32 |
| Multivariable 1 | 1.53 (0.91, 2.57) | 1.02 (0.8, 1.28) | ref | 1.13 (0.64, 1.98) | 0.48 |
| Multivariable 2 | 1.47 (0.87, 2.47) | 0.99 (0.78, 1.25) | ref | 1.15 (0.65, 2.02) | 0.62 |
| Brain Cancer |  |  |  |  |  |
| No. of cases | 10 | 148 | 307 | 15 |  |
| Age adjusted | 0.72 (0.38, 1.34) | 0.96 (0.79, 1.16) | ref | 0.89 (0.53, 1.50) | 0.51 |
| Multivariable 1 | 0.78 (0.41, 1.47) | 0.99 (0.81, 1.21) | ref | 0.89 (0.53, 1.50) | 0.82 |
| Multivariable 2 | 0.77 (0.41, 1.45) | 0.98 (0.8, 1.19) | ref | 0.92 (0.55, 1.55) | 0.58 |
| Non-Hodgkin Lymphoma |  |  |  |  |  |
| No. of cases | 38 | 563 | 1043 | 68 |  |
| Age adjusted | 0.81 (0.58, 1.11) | 1.08 (0.97, 1.19) | ref | 1.18 (0.92, 1.51) | 0.92 |
| Multivariable 1 | 0.86 (0.62, 1.19) | 1.1 (0.99, 1.22) | ref | 1.19 (0.93, 1.52) | 0.70 |
| Multivariable 2 | 0.83 (0.60, 1.15) | 1.09 (0.98, 1.21) | ref | 1.19 (0.93, 1.52) | 0.80 |
| Leukemia |  |  |  |  |  |
| No. of cases | 24 | 314 | 583 | 43 |  |
| Age adjusted | 0.92 (0.61, 1.38) | 1.08 (0.94, 1.24) | ref | 1.32 (0.97, 1.80) | 0.94 |
| Multivariable 1 | 0.97 (0.64, 1.47) | 1.12 (0.97, 1.28) | ref | 1.29 (0.95, 1.77) | 0.69 |
| Multivariable 2 | 0.95 (0.63, 1.43) | 1.11 (0.96, 1.27) | ref | 1.29 (0.94, 1.76) | 0.73 |
| Myeloma |  |  |  |  |  |
| No. of cases | 24 | 160 | 319 | 16 |  |
| Age adjusted | 1.68 (1.11, 2.54) | 1.01 (0.83, 1.22) | ref | 0.90 (0.54, 1.48) | 0.15 |
| Multivariable 1 | 1.69 (1.11, 2.59) | 1.01 (0.84, 1.23) | ref | 0.89 (0.54, 1.47) | 0.14 |
| Multivariable 2 | 1.65 (1.08, 2.52) | 1.01 (0.83, 1.22) | ref | 0.88 (0.53, 1.46) | 0.11 |
| Breast Cancer^*^ |  |  |  |  |  |
| No. of cases | 162 | 1920 | 3648 | 189 |  |
| Age adjusted | 0.76 (0.65, 0.89) | 0.95 (0.90, 1.01) | ref | 0.93 (0.80, 1.07) | 0.007 |
| Multivariable 1 | 0.80 (0.68, 0.94) | 0.98 (0.92, 1.03) | ref | 0.91 (0.79, 1.06) | 0.130 |
| Multivariable 2 | 0.80 (0.68, 0.93) | 0.98 (0.93, 1.04) | ref | 0.90 (0.77, 1.04) | 0.22 |
| Ovarian Cancer^*^ |  |  |  |  |  |
| No. of cases | 13 | 175 | 318 | 9 |  |
| Age adjusted | 0.70 (0.40, 1.21) | 1.00 (0.83, 1.20) | ref | 0.50 (0.26, 0.98) | 0.86 |
| Multivariable 1 | 0.76 (0.43, 1.32) | 1.04 (0.86, 1.25) | ref | 0.51 (0.26, 0.99) | 0.52 |
| Multivariable 2 | 0.76 (0.44, 1.34) | 1.04 (0.86, 1.25) | ref | 0.51 (0.26, 0.99) | 0.47 |
| Endometrial Cancer ^*^ |  |  |  |  |  |
| No. of cases | 37 | 240 | 492 | 37 |  |
| Age adjusted | 1.26 (0.94, 1.70) | 0.87 (0.76, 0.99) | ref | 1.24 (0.91, 1.68) | 0.19 |
| Multivariable 1 | 1.24 (0.92, 1.68) | 0.88 (0.77, 1.01) | ref | 1.16 (0.85, 1.58) | 0.32 |
| Multivariable 2 | 1.16 (0.86, 1.58) | 0.87 (0.75, 0.99) | ref | 1.13 (0.83, 1.54) | 0.38 |
| Prostate Cancer^**^ |  |  |  |  |  |
| No. of cases | 299 | 4076 | 9199 | 470 |  |
| Age adjusted | 0.91 (0.81, 1.02) | 0.96 (0.92, 0.99) | ref | 0.93 (0.85, 1.02) | 0.04 |
| Multivariable 1 | 0.96 (0.85, 1.07) | 0.97 (0.93, 1.00) | ref | 0.96 (0.88, 1.06) | 0.16 |
| Multivariable 2 | 0.96 (0.86, 1.08) | 0.97 (0.94, 1.01) | ref | 0.96 (0.87, 1.05) | 0.27 |

Cox proportional hazard model was used to calculate hazard ratios. Multivariable 1removed physical activity, sedentary behavior, BMI, diabetes, hypertension, or any dietary variables from the multivariable 2. Multivariable 2 adjusted for age, gender, napping, race, education, marital status, self-reported health, family history of cancer, smoking (former/current/never, as well as dose and years after quitting), physical activity, sitting time, diabetes, hypertension, body mass index, NSAID use, alcohol drinking, intakes of fruits and vegetables, wholegrain, total fat, red meat and total calories. ^*^ For female cancers the multivariable model additionally adjusted for postmenopausal hormonal use, menopausal status, number of live child birth, oral contraception use, hysterectomy and oophorectomy. ^**^For prostate cancer, we additionally adjusted for PSA screening.
