# Supplementary material for: Sleep Duration and Cancer in the NIH-AARP Diet and Health Study Cohort
Source: PLoS One. 2016 Sep 9;11(9):e0161561. doi: 10.1371/journal.pone.0161561 (PMC5017779; doi:10.1371/journal.pone.0161561)
Supplement: S2 Table — Cox proportional hazard model was used to calculate hazard ratios. Model adjusted for age, gender, napping, race, education, marital status, self-reported health, family history of cancer, smoking (former/current/never, as well as dose and years after quitting), physical activity, sitting time, diabetes, hypertension, body mass index, NSAID use, alcohol drinking, intakes of fruits and vegetables, wholegrain, total fat, red meat and total calories. * For female cancers model additionally adjusted for postmenopausal hormonal use, menopausal status, number of live child birth, oral contraception use, hysterectomy and oophorectomy. (DOC) [file pone.0161561.s002.doc]

**S2 Table. Sensitivity analyses by excluding cancer cases diagnosed within 2 years of enrollment for selected sites**

|  | **Hazard Ratio (95% Confidence Interval)** | | | |  |
| --- | --- | --- | --- | --- | --- |
|  | **Sleep at night < 5 hrr** | **5-6 hr** | **7-8 hr** | **≥ 9 hr** | ***P* _trend_** |
| Head and Neck cancer |  |  |  |  |  |
| No. of cases after exclusion | 45 | 327 | 638 | 46 |  |
| Before exclusion | 1.33 (1.00, 1.78) | 1.08 (0.95, 1.21) | Ref | 1.10 (0.84, 1.45) | *0.17* |
| After exclusion | 1.49 (1.10, 2.04) | 1.04 (0.9, 1.19) | ref | 1.16 (0.86, 1.56) | *0.3* |
| Stomach Cancer |  |  |  |  |  |
| No. of cases after exclusion | 11 | 154 | 231 | 11 |  |
| Before exclusion | 0.87 (0.51, 1.50) | 1.24 (1.03, 1.49) | Ref | 0.75 (0.44, 1.29) | *0.04* |
| After exclusion | 0.86 (0.47, 1.59) | 1.27 (1.03, 1.56) | ref | 0.76 (0.42, 1.40) | *0.05* |
| Myeloma |  |  |  |  |  |
| No. of cases after exclusion | 20 | 136 | 273 | 12 |  |
| Before exclusion | 1.65 (1.08, 2.52) | 1.01 (0.83, 1.22) | ref | 0.88 (0.53, 1.46) | *0.11* |
| After exclusion | 1.57 (0.98, 2.49) | 0.99 (0.80, 1.21) | ref | 0.78 (0.44, 1.40) | *0.21* |
| Breast Cancer^*^ |  |  |  |  |  |
| No. of cases after exclusion | 124 | 1526 | 2864 | 146 |  |
| Before exclusion | 0.80 (0.68, 0.93) | 0.98 (0.93, 1.04) | ref | 0.90 (0.77, 1.04) | *0.22* |
| After exclusion | 0.80 (0.67, 0.96) | 1.00 (0.94, 1.06) | ref | 0.89 (0.75, 1.05) | *0.52* |
| Ovarian Cancer^*^ |  |  |  |  |  |
| No. of cases after exclusion | 12 | 145 | 228 | 6 |  |
| Before exclusion | 0.76 (0.44, 1.34) | 1.04 (0.86, 1.25) | ref | 0.51 (0.26, 0.99) | *0.47* |
| After exclusion | 0.97 (0.54, 1.75) | 1.19 (0.96, 1.47) | ref | 0.48 (0.22, 1.09) | *0.05* |
| Endometrial Cancer^*^ |  |  |  |  |  |
| No. of cases after exclusion | 47 | 304 | 635 | 44 |  |
| Before exclusion | 1.16 (0.86, 1.58) | 0.87 (0.75, 0.99) | ref | 1.13 (0.83, 1.54) | *0.38* |
| After exclusion | 1.22 (0.87, 1.72) | 0.88 (0.76, 1.03) | ref | 1.26 (0.90, 1.77) | *0.45* |

Cox proportional hazard model was used to calculate hazard ratios. Model adjusted for age, gender, napping, race, education, marital status, self-reported health, family history of cancer, smoking (former/current/never, as well as dose and years after quitting), physical activity, sitting time, diabetes, hypertension, body mass index, NSAID use, alcohol drinking, intakes of fruits and vegetables, wholegrain, total fat, red meat and total calories. ^*^ For female cancers model additionally adjusted for postmenopausal hormonal use, menopausal status, number of live child birth, oral contraception use, hysterectomy and oophorectomy.
