# Supplementary material for: Sleep Duration and Cancer in the NIH-AARP Diet and Health Study Cohort
Source: PLoS One. 2016 Sep 9;11(9):e0161561. doi: 10.1371/journal.pone.0161561 (PMC5017779; doi:10.1371/journal.pone.0161561)
Supplement: S3 Table — Cox proportional hazard model was used to calculate hazard ratios. Model adjusted for age, gender, napping, race, education, marital status, self-reported health, family history of cancer, smoking (former/current/never, as well as dose and years after quitting), physical activity, sitting time, diabetes, hypertension, body mass index, NSAID use, alcohol drinking, intakes of fruits and vegetables, wholegrain, total fat, red meat and total calories. For breast cancer, the model additionally adjusted for postmenopausal hormonal use, menopausal status, number of live child birth, oral contraception use, hysterectomy and oophorectomy. For prostate cancer, we additionally adjusted for PSA screening. (DOC) [file pone.0161561.s003.doc]

**S3 Table. Sensitivity analyses for cancer sites with case number > 4000**

|  | **Hazard Ratio (95% Confidence Interval)** | | | |  |
| --- | --- | --- | --- | --- | --- |
|  | **Sleep at night < 5 hr** | **5-6 hr** | **7-8 hr** | **≥ 9 hr** | ***P* _trend_** |
| Colorectal Cancer |  |  |  |  |  |
| No diabetes | 0.97 (0.80, 1.18) | 1.00 (0.93, 1.07) | ref | 1.11 (0.94, 1.30) | *0.56* |
| No "poor" health | 0.95 (0.79, 1.14) | 1.02 (0.95, 1.09) | ref | 1.10 (0.95, 1.29) | *0.71* |
| By body mass index |  |  |  |  |  |
| Normal | 1.07 (0.78, 1.48) | 1.00 (0.89, 1.12) | ref | 1.12 (0.86, 1.45) | *0.82* |
| Overweight | 0.92 (0.74, 1.15) | 1.03 (0.95, 1.11) | ref | 1.13 (0.94, 1.35) | *0.64* |
| By physical activity |  |  |  |  |  |
| <=3 hr/wk | 1.01 (0.80, 1.28) | 1.04 (0.95, 1.13) | ref | 0.94 (0.76, 1.17) | *0.34* |
| >3 hr/wk | 0.91 (0.67, 1.21) | 1.01 (0.91, 1.11) | ref | 1.38 (1.12, 1.71) | *0.16* |
| By napping |  |  |  |  |  |
| no | 0.94 (0.70, 1.27) | 1.03 (0.93, 1.14) | ref | 1.16 (0.93, 1.44) | *0.77* |
| yes | 0.97 (0.78, 1.23) | 1.01 (0.92, 1.1) | ref | 1.09 (0.89, 1.34) | *0.82* |
| Lung Cancer |  |  |  |  |  |
| No diabetes | 0.93 (0.79, 1.09) | 1.02 (0.96, 1.08) | ref | 1.00 (0.86, 1.15) | *0.68* |
| No "poor" health | 0.91 (0.78, 1.07) | 1.02 (0.96, 1.08) | ref | 0.96 (0.84, 1.11) | *0.86* |
| By body mass index |  |  |  |  |  |
| Normal | 0.99 (0.78, 1.26) | 1.04 (0.95, 1.13) | ref | 1.11 (0.91, 1.35) | *0.95* |
| Overweight | 0.88 (0.72, 1.07) | 1.01 (0.94, 1.09) | ref | 0.84 (0.69, 1.02) | *0.78* |
| By physical activity |  |  |  |  |  |
| <=3 hr/wk | 0.94 (0.77, 1.15) | 1.06 (0.98, 1.15) | ref | 0.89 (0.74, 1.07) | *0.3* |
| >3 hr/wk | 0.87 (0.68, 1.12) | 0.98 (0.9, 1.08) | ref | 1.08 (0.87, 1.34) | *0.23* |
| By napping |  |  |  |  |  |
| no | 0.69 (0.52, 0.93) | 1.02 (0.93, 1.11) | ref | 1.17 (0.97, 1.41) | *0.07* |
| yes | 1.03 (0.86, 1.23) | 1.02 (0.94, 1.10) | ref | 0.77 (0.63, 0.95) | *0.19* |
| Breast Cancer |  |  |  |  |  |
| No diabetes | 0.80 (0.68, 0.95) | 0.99 (0.93, 1.05) | ref | 0.89 (0.77, 1.04) | *0.37* |
| No "poor" health | 0.81 (0.69, 0.95) | 0.98 (0.92, 1.04) | ref | 0.91 (0.78, 1.05) | *0.22* |
| By body mass index |  |  |  |  |  |
| Normal | 0.78 (0.60, 1.03) | 0.95 (0.88, 1.04) | ref | 1.01 (0.82, 1.24) | *0.08* |
| Overweight | 0.81 (0.66, 0.99) | 1.00 (0.93, 1.08) | ref | 0.81 (0.65, 0.99) | *0.87* |
| By physical activity |  |  |  |  |  |
| <=3 hr/wk | 0.80 (0.64, 1.00) | 0.98 (0.91, 1.06) | ref | 0.84 (0.68, 1.02) | *0.63* |
| >3 hr/wk | 0.83 (0.66, 1.06) | 0.97 (0.89, 1.05) | ref | 0.97 (0.78, 1.20) | *0.23* |
| By napping |  |  |  |  |  |
| no | 0.83 (0.66, 1.03) | 0.99 (0.91, 1.06) | ref | 0.90 (0.74, 1.10) | *0.52* |
| yes | 0.77 (0.62, 0.97) | 0.97 (0.89, 1.05) | ref | 0.89 (0.71, 1.11) | *0.26* |
| Prostate Cancer |  |  |  |  |  |
| No diabetes | 0.96 (0.84, 1.08) | 0.98 (0.94, 1.01) | ref | 0.96 (0.87, 1.06) | *0.29* |
| No "poor" health | 0.95 (0.85, 1.07) | 0.97 (0.94, 1.01) | ref | 0.96 (0.87, 1.05) | *0.23* |
| By body mass index |  |  |  |  |  |
| Normal | 1.06 (0.85, 1.31) | 1.00 (0.93, 1.06) | ref | 1.04 (0.90, 1.22) | *0.88* |
| Overweight | 0.93 (0.81, 1.07) | 0.96 (0.92, 1.01) | ref | 0.91 (0.81, 1.03) | *0.27* |
| By physical activity |  |  |  |  |  |
| <=3 hr/wk | 0.88 (0.74, 1.04) | 0.99 (0.93, 1.04) | ref | 0.95 (0.83, 1.08) | *0.39* |
| >3 hr/wk | 1.04 (0.88, 1.22) | 0.96 (0.91, 1.01) | ref | 0.96 (0.84, 1.10) | *0.37* |
| By napping |  |  |  |  |  |
| no | 1.03 (0.85, 1.25) | 1.00 (0.95, 1.06) | ref | 0.99 (0.87, 1.14) | *0.99* |
| yes | 0.92 (0.8, 1.07) | 0.95 (0.91, 1.00) | ref | 0.93 (0.81, 1.05) | *0.12* |

Cox proportional hazard model was used to calculate hazard ratios. Model adjusted for age, gender, napping, race, education, marital status, self-reported health, family history of cancer, smoking (former/current/never, as well as dose and years after quitting), physical activity, sitting time, diabetes, hypertension, body mass index, NSAID use, alcohol drinking, intakes of fruits and vegetables, wholegrain, total fat, red meat and total calories. For breast cancer, the model additionally adjusted for postmenopausal hormonal use, menopausal status, number of live child birth, oral contraception use, hysterectomy and oophorectomy. For prostate cancer, we additionally adjusted for PSA screening.
